# Left frontal glioma induces functional connectivity changes in syntax-related networks

**DOI:** 10.1186/s40064-015-1104-6

**Published:** 2015-07-04

**Authors:** Ryuta Kinno, Shinri Ohta, Yoshihiro Muragaki, Takashi Maruyama, Kuniyoshi L Sakai

**Affiliations:** Department of Basic Science, Graduate School of Arts and Sciences, The University of Tokyo, 3-8-1 Komaba, Meguro-ku, Tokyo, 153-8902 Japan; Division of Neurology, Department of Internal Medicine, Showa University Northern Yokohama Hospital, 35-1 Chigasaki-chuo, Tsuzuki-ku, Yokohama, Kanagawa, 224-8503 Japan; CREST, Japan Science and Technology Agency, 7 Gobancho, Chiyoda-ku, Tokyo, 102-0076 Japan; Department of Neurosurgery, Tokyo Women’s Medical University, 8-1 Kawada-cho, Shinjuku-ku, Tokyo, 162-8666 Japan

**Keywords:** Agrammatic comprehension, Frontal regions, Functional connectivity, Functional MRI, Glioma, Neural network

## Abstract

**Background:**

A glioma leads to a global loss of functional connectivity among multiple regions. However, the relationships between performance/activation changes and functional connectivity remain unclear. Our previous studies (Brain 137:1193–1212; Brain Lang 110:71–80) have shown that a glioma in the left lateral premotor cortex or the opercular/triangular parts of the left inferior frontal gyrus causes agrammatic comprehension accompanied by abnormal activations in 14 syntax-related regions. We have also confirmed that a glioma in the *other* left frontal regions does not affect task performances and activation patterns.

**Results:**

By a partial correlation method for the time-series functional magnetic resonance imaging data, we analyzed the functional connectivity in 21 patients with a left frontal glioma. We observed that almost all of the functional connectivity exhibited chaotic changes in the agrammatic patients. In contrast, some functional connectivity was preserved in an orderly manner in the patients who showed normal performances and activation patterns. More specifically, these latter patients showed normal connectivity between the left fronto-parietal regions, as well as normal connectivity between the left triangular and orbital parts of the left inferior frontal gyrus.

**Conclusions:**

Our results indicate that these pathways are most crucial among the syntax-related networks. Both data from the activation patterns and functional connectivity, which are different in temporal domains, should thus be combined to assess any behavioral deficits associated with brain abnormalities.

**Electronic supplementary material:**

The online version of this article (doi:10.1186/s40064-015-1104-6) contains supplementary material, which is available to authorized users.

## Findings

### Background

Gliomas are generally considered to cause hyperexcitability throughout the entire brain, leading to epileptic seizures (de Groot et al. 2012). A magnetoencephalogram study of the resting-state functional connectivity showed that a glioma disrupted functional connectivity across not only the regions it occupied, but also other distant regions in both hemispheres of the cerebral cortex (Bartolomei et al. 2006). A recent functional magnetic resonance imaging (fMRI) study with a verb-generation task reported that a single glioma partially reduced the functional connectivity among the bilateral frontal and temporal regions (Briganti et al. 2012). In such lesion studies, however, not only functional connectivity but activation patterns should be clarified, because their relationships may depend on any tasks and neural networks involved.

We have previously shown that a glioma in the left lateral premotor cortex (LPMC) or in the opercular/triangular parts of the left inferior frontal gyrus (F3op/F3t) is sufficient to cause agrammatic comprehension (Kinno et al. 2009). Moreover, those agrammatic patients showed abnormal task-related activations in 14 regions (Table 1). The behavioral data and activation maps obtained from the normal controls (Normal group) and patients have been reported in our previous paper (Kinno et al. 2014). More specifically, patients with a glioma in the left LPMC (LPMC group) had a more profound deficit in the comprehension of scrambled sentences than that of active and passive sentences, while patients with a glioma in the left F3op/F3t (F3 group) had a more profound deficit in the comprehension of passive and scrambled sentences than that of active sentences. Furthermore, the LPMC and F3 groups showed abnormal overactivity and/or underactivity in the 14 regions, while the patients with a glioma in the other left frontal regions (Other group) showed normal performances and activation patterns. These 14 regions were grouped into three syntax-related networks: Network I consists of the left F3op/F3t, left intraparietal sulcus (IPS), right frontal regions, pre-supplementary motor area, and right temporal regions. Network II consists of the left LPMC, left angular gyrus (AG), lingual gyrus, and cerebellar nuclei. Network III consists of the left ventral frontal and posterior temporal regions. Some functional connectivity may be preserved in the Other group, and we infer that the identification of such preserved connectivity would reveal the most crucial pathways among the syntax-related networks, which has not been possible by the study of normal controls alone. The present study thus underscores the importance of examining functional connectivity changes in brain-damaged patients.

**Table 1:** Brain regions within three syntax-related networks

Brain region	BA	Side	*x*	*y*	*z*
Network I					
F3op/F3t	44/45	L	–45	18	27
IPS	7/39/40	L	–21	–72	51
LPMC	6/8	R	30	3	45
F3op/F3t	44/45	R	33	18	24
pre-SMA	6/8	M	9	24	51
pSTG/MTG	22/21	R	60	–57	3
Network II					
LPMC	6/8	L	–48	3	42
AG	39	L	–33	–60	18
LG	18	M	–3	–69	6
Cerebellar nuclei		M	–3	–51	–27
Network III					
F3t	45	L	–48	33	6
F3O	47	L	–36	15	–6
pSTG/MTG	22/21	L	–57	–48	0
pMTG/ITG	37/19	L	–45	–69	0

Stereotactic coordinates (*x, y, z*) in the Montreal Neurological Institute space are shown for 14 syntax-related regions, functionally determined in our previous study (Kinno et al. 2014). All but two regions were within the gray matter; the right LPMC and left AG were on the border of the gray matter.

*AG* angular gyrus, *BA* Brodmann’s area, *F3O* orbital part of the inferior frontal gyrus, *F3op/F3t* opercular/triangular parts of the inferior frontal gyrus, *F3t* triangular part of the inferior frontal gyrus, *IPS* intraparietal sulcus, *L* left, *LG* lingual gyrus, *LPMC* lateral premotor cortex, *M* medial, *pMTG/ITG* posterior middle/inferior temporal gyri, *pre-SMA* pre-supplementary motor area, *pSTG/MTG* posterior superior/middle temporal gyri, *R* right.

## Methods

Here, we provided an overview of the methods; full details are provided in an additional file (see Additional file 1). We analyzed the functional connectivity in 21 patients (Table 2) and 7 normal participants reported previously (Kinno et al. 2014). The fMRI data were analyzed in a standard manner using statistical parametric mapping (SPM8) software with the “unified segmentation” algorithm, which is a generative model that combines tissue segmentation (excluding “other” tissues like a lesion, etc.), bias correction, and spatial normalization (Ashburner and Friston 2005). All normalized functional images were then smoothed by using an isotropic Gaussian kernel of 9 mm full-width at half maximum. Low-frequency noise was removed by high-pass filtering at 1/128 Hz. For creating an SPM design of each participant, hemodynamic responses induced by task trials were modeled with a boxcar function for 6 s from the onset of each stimulus, and the boxcar function was convolved with a hemodynamic response function. The functional connectivity among multiple regions was assessed by a partial correlation method for the time-series fMRI data (Smith 2012). Using the MarsBaR-toolbox (http://marsbar.sourceforge.net/) for the SPM design, the time-series data were first averaged within a sphere of 6-mm radius centered at the local maximum of each region (Table 1). To discount the global differences of signal changes among the runs, the averaged time-series from each of all tested runs were normalized to those of the first run. Using the concatenated runs of each participant, we calculated partial correlation coefficients for each of the time-series of two regions in question; we regressed out all the other nodes, before estimating the correlation between the two. The partial correlation coefficients were then averaged among the participants in each group to generate a partial correlation matrix.

**Table 2:** Patient demographics

Patient	Age	Laterality quotient	Tumor location	Tumor type	Tumor grade
LPMC group					
Patient 1	31	88	L. F1/F2/SMA/LPMC	AO	III
Patient 2	47	81	L. F1/F2/SMA/LPMC/F3op	AOA	III
Patient 3	36	88	L. F1/F2/SMA/LPMC/F3op	AA	III
Patient 4	49	100	L. F1/F2/LPMC	AO	III
Patient 5	34	100	L. F1/F2/LPMC/F3op	OD	II
Patient 6	29	81	L. F1/F2/LPMC/F3op	AO	III
Patient 7	27	45	L. F2/LPMC/F3op	DA	II
Mean ± SD	36 ± 8.6	83 ± 19			
F3 group					
Patient 8	32	54	L. F1/F2/F3op/F3O/insula	AOA	III
Patient 9	20	100	L. F1/F2/F3op/F3O/insula	AO	III
Patient 10	31	100	L. F1/F2/F3op/F3t/insula/striatum	AOA	III
Patient 11	32	100	L. F1/F2/F3op/F3O/insula/striatum	DA	II
Patient 12	42	90	L. F2/F3op/F3O/insula/striatum	OA	II
Patient 13	29	87	L. F2/F3op/F3O/insula/striatum	OD	II
Patient 14	47	73	L. F3op/F3t/insula/striatum	DA	II
Mean ± SD	33 ± 8.8	86 ± 17			
Other group					
Patient 15	62	89	L. F1/F2/SMA	AO	III
Patient 16	24	68	L. F1/F2/SMA/striatum	OA	II
Patient 17	38	100	L. F1/F2/SMA/striatum	AOA	III
Patient 18	21	100	L. F1/F2/F3t/striatum	DA	II
Patient 19	29	100	L. F1/F2/F3O/striatum	AO	III
Patient 20	25	100	L. F1/F2/F3t	DA	II
Patient 21	36	100	L. F2/F3O	OA	II
Mean ± SD	34 ± 14	83 ± 12			

The laterality quotient of handedness was determined by the Edinburgh handedness inventory (Oldfield 1971). The tumor type and grade were postoperatively and pathologically diagnosed by the World Health Organization Classification of Tumors of the Nervous System (2000).

*AA* anaplastic astrocytoma (grade III), *AO* anaplastic oligodendroglioma (grade III), *AOA* anaplastic oligoastrocytoma (grade III), *DA* diffuse astrocytoma (grade II), *F1* superior frontal gyrus, *F2* middle frontal gyrus, *F3O* orbital part of the inferior frontal gyrus, *F3op* opercular part of the inferior frontal gyrus, *F3t* triangular part of the inferior frontal gyrus, *L.* left, *LPMC* lateral premotor cortex, *OA* oligoastrocytoma (grade II), *OD* oligodendroglioma (grade II), *SD* standard deviation, *SMA* supplementary motor area.

## Results

**Figure 1:**
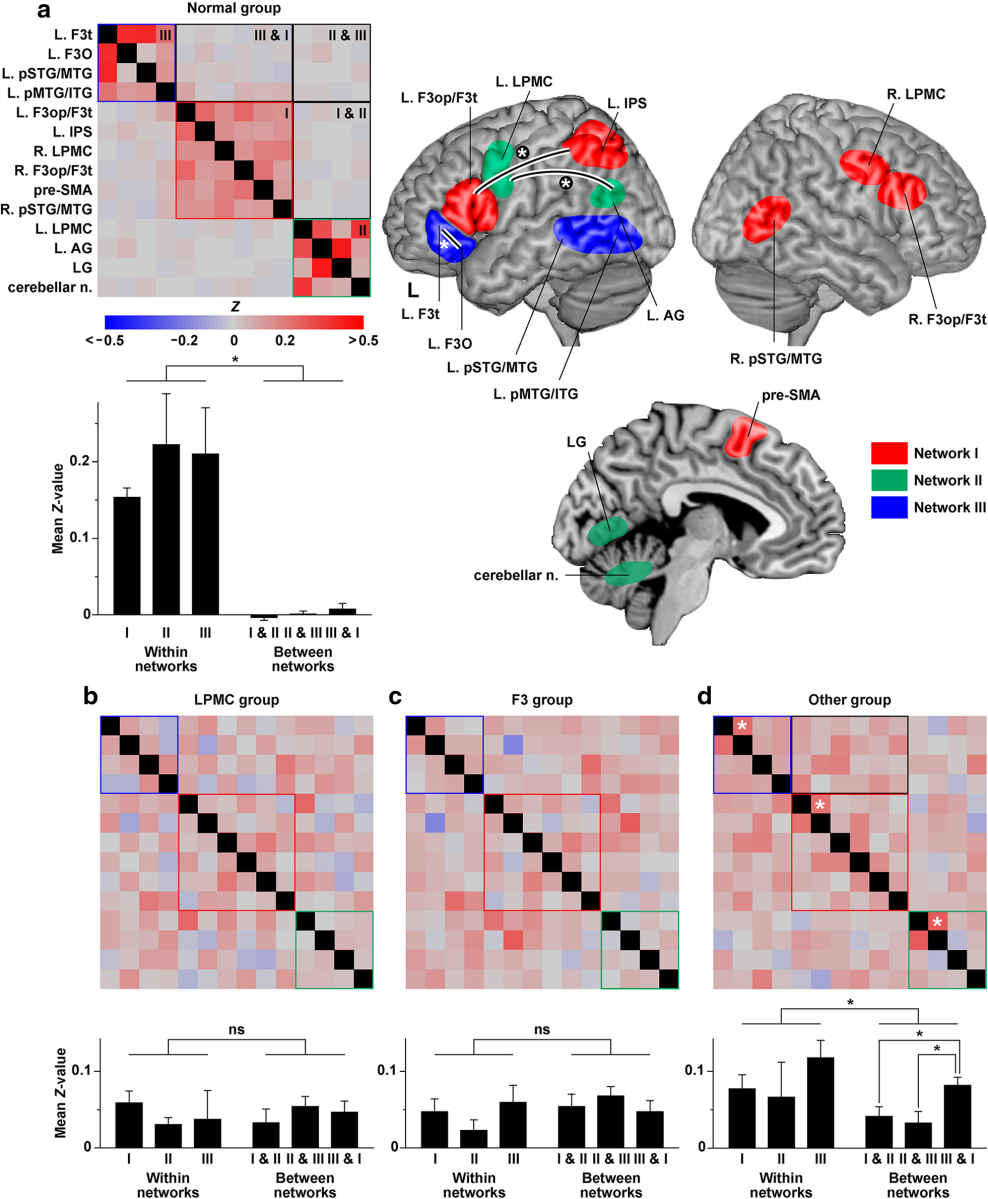
Orderly or chaotic functional connectivity within syntax-related networks. **a** Normal functional connectivity within syntax-related networks. A partial correlation matrix and mean *Z* values within/between the three networks are shown for the Normal group, together with a schematic of the three syntax-related networks (I, II, and III) in the *right panel*. In the partial correlation matrix, region pairs of “within networks” surrounded by *red*, *green*, and *blue boxes* correspond to Networks I, II, and III, respectively, whereas region pairs of “between networks” are surrounded by a *black box* for each network pair. All diagonal elements were shown in *black*. The *r* values reported in the previous study (Kinno et al. 2014) were here converted to *Z* values by using the Fisher *r*-to-*Z* transformation. *Error bars* in the bar graphs indicate the SEM for the multiple region pairs. In the schematic, the brain regions of Networks I, II, and III are shown in *red,* *green*, and *blue*, respectively. **b**–**d** A partial correlation matrix and mean *Z* values within/between the three networks in the LPMC (**b**), F3 (**c**), and Other (**d**) groups (novel results in the present study). Each of the *three white asterisks* in the matrix (**d**) indicates a higher level of functional connectivity significantly different from the abnormal connectivity (*P* < 0.05). These three connections are also denoted by *curved lines* and *white asterisks* in the schematic (**a**). In the matrix (**d**), a network pair of Networks III & I surrounded by a *black box* shows increased connectivity compared to the other network pairs. *AG* angular gyrus, *F3O* orbital part of the inferior frontal gyrus, *F3op/F3t* opercular/triangular parts of the inferior frontal gyrus, *F3t* triangular part of the inferior frontal gyrus, *IPS* intraparietal sulcus, *L.* left, *LG* lingual gyrus, *LPMC* lateral premotor cortex, *n.* nuclei, *pMTG/ITG* posterior middle/inferior temporal gyri, *pre-SMA* pre-supplementary motor area, *pSTG/MTG* posterior superior/middle temporal gyri, *R.* right.

The partial correlation matrix for the Normal group clearly demonstrated the existence of three separate syntax-related networks (Figure 1a). Indeed, all of the functional connectivity within those networks was positive (mean *Z* value, 0.18), while “cross-talk” between these networks was absent for the Normal group (mean *Z* value, 0.002). We defined a “network-boundary effect” as the significantly greater connectivity among the regions within individual networks compared with other connectivity. The mean *Z* values within individual networks were significantly greater than those between any two of the networks (one-tailed *t* test, *P* < 0.0001), confirming the network-boundary effects.

According to the partial correlation matrix for the LPMC and F3 groups, almost all of the functional connectivity became abnormal, including that of the networks outside lesions (Figure 1b, c). Moreover, the network-boundary effects completely disappeared for both patient groups (*P* > 0.3). Based on the comparison of mean *Z* values between each patient group and Normal group, both patient groups showed significantly *decreased* connectivity within individual networks (Dunnett test, *P* < 0.0001), together with significantly *increased* connectivity between any two of the networks (*P* < 0.0003). Given such a leveling effect, the *Z* values averaged among all region pairs were 0.045 and 0.052 for the LPMC and F3 groups, respectively; we regarded the higher *Z* value of 0.052 as a typical value for abnormal connectivity.

According to the partial correlation matrix for the Other group (Figure 1d), some normal functional connectivity was preserved in the following region pairs (the first two regions listed for each network; see Table 1): the left F3op/F3t and left IPS (*Z* = 0.22), the left LPMC and left AG (*Z* = 0.28), and the triangular and orbital parts of the left inferior frontal gyri (F3t and F3O) (*Z* = 0.25). Within individual networks, only these *Z* values were significantly above the abnormal connectivity at *Z* = 0.052 (one-tailed one-sample *t* test among the Other group, *P* < 0.03). While the network-boundary effects were significant for the Other group (*P* = 0.026), this group showed significantly *decreased* connectivity within individual networks compared to the Normal group (Dunnett test, *P* < 0.0001), together with significantly *increased* connectivity between any two of the networks (*P* < 0.0001). In addition, this group showed significantly increased connectivity between Networks III and I compared to the other network pairs (Tukey–Kramer test, *P* < 0.05).

## Discussion

We showed that the network-boundary effects were significant for both the Normal and Other groups (Figure 1a, d), while almost all of the functional connectivity exhibited chaotic changes in the LPMC and F3 groups (Figure 1b, c). It was notable that some functional connectivity was preserved in an orderly manner in the Other group, who showed normal task performances and activation patterns. More specifically, the Other group showed normal functional connectivity between the left F3op/F3t and left IPS (Network I), between the left LPMC and left AG (Network II), and between the left F3t and left F3O (Network III). Our results indicate that these pathways are most crucial among the syntax-related networks.

The crucial pathways within Networks I and II (Figure 1a), which were preserved in the Other group, actually pass through a dorsal pathway (i.e., the arcuate fasciculus and superior longitudinal fasciculus) in the left hemisphere. Some previous fMRI and diffusion MRI studies have suggested the importance of the dorsal pathway for syntactic processing (Friederici et al. 2006; Saur et al. 2008; Ohta et al. 2013). As regards the ventral pathway connecting the left F3t and left F3O, a recent study with fMRI and diffusion MRI has suggested that these regions make a triangulo-orbitaris system connected by intermingled U-shaped fibers (Lemaire et al. 2013). The integrity of both the dorsal and ventral pathways would thus be related to normal performances. The increased connectivity between Network III and I in the Other group may reflect rescuing interactions among these syntax-related networks to achieve normal syntactic processing.

We observed the leveling effect in all three of the patient groups (Figure 1b–d). We assume that the loss of functional connectivity observed in the present study reflects the globally—i.e., without any specific correlates of cognitive processes—increased excitability due to the upregulation of intrinsically bursting neurons within the peritumoral zone (de Groot et al. 2012). In spite of such abnormal excitability in the entire brain, it is surprising to note that both the LPMC and F3 groups showed distinct overactivity and/or underactivity among the regions within each of Networks I–III (Kinno et al. 2014). Moreover, these patient groups showed the different types of agrammatic comprehension, as well as differential activation patterns, although their abnormal functional connectivity was essentially similar. Furthermore, the Other group showed normal performances and activation patterns, even if functional connectivity was partially abnormal. Therefore, the global loss of functional connectivity may not be the primary cause of the abnormal performances and activation patterns; rather the loss of connectivity must be the complication of the glioma. The comparison of data between normal controls and patients would thus provide clinically valuable information, such as preserved functional connectivity and relevant functions. We believe that diagnoses utilizing such information would be effective to medical treatment including rehabilitation for intractable cognitive or behavioral deficits.

## Additional file

**Additional file 1.** Supplementary Methods.

## References

[CR1] Ashburner J, Friston KJ (2005). Unified segmentation. Neuroimage.

[CR2] Bartolomei F, Bosma I, Klein M, Baayen JC, Reijneveld JC, Postma TJ (2006). How do brain tumors alter functional connectivity ? A magnetoencephalography study. Ann Neurol.

[CR3] Briganti C, Sestieri C, Mattei PA, Esposito R, Galzio RJ, Tartaro A (2012). Reorganization of functional connectivity of the language network in patients with brain gliomas. Am J Neuroradiol.

[CR4] de Groot M, Reijneveld JC, Aronica E, Heimans JJ (2012). Epilepsy in patients with a brain tumour: Focal epilepsy requires focused treatment. Brain.

[CR5] Friederici AD, Bahlmann J, Heim S, Schubotz RI, Anwander A (2006). The brain differentiates human and non-human grammars: Functional localization and structural connectivity. Proc Natl Acad Sci USA.

[CR6] Kinno R, Muragaki Y, Hori T, Maruyama T, Kawamura M, Sakai KL (2009). Agrammatic comprehension caused by a glioma in the left frontal cortex. Brain Lang.

[CR7] Kinno R, Ohta S, Muragaki Y, Maruyama T, Sakai KL (2014). Differential reorganization of three syntax-related networks induced by a left frontal glioma. Brain.

[CR8] Lemaire J-J, Golby A, Wells WM, Pujol S, Tie Y, Rigolo L (2013). Extended Broca’s area in the functional connectome of language in adults: Combined cortical and subcortical single-subject analysis using fMRI and DTI tractography. Brain Topogr.

[CR9] Ohta S, Fukui N, Sakai KL (2013). Syntactic computation in the human brain: The Degree of Merger as a key factor. PLOS One.

[CR10] Oldfield RC (1971). The assessment and analysis of handedness: The Edinburgh inventory. Neuropsychologia.

[CR11] Saur D, Kreher BW, Schnell S, Kümmerer D, Kellmeyer P, Vry M-S (2008). Ventral and dorsal pathways for language. Proc Natl Acad Sci USA.

[CR12] Smith SM (2012). The future of FMRI connectivity. Neuroimage.

