# Supplementary material for: Left frontal glioma induces functional connectivity changes in syntax-related networks
Source: Springerplus. 2015 Jul 4;4:317. doi: 10.1186/s40064-015-1104-6 (PMC4491091; doi:10.1186/s40064-015-1104-6)
Supplement: Supplementary file 1 — Additional file 1. Supplementary Methods. [file 40064_2015_1104_MOESM1_ESM.pdf]

Additional file 1

Left frontal glioma induces functional connectivity changes in syntax-related networks

Ryuta Kinno, Shinri Ohta, Yoshihiro Muragaki, Takashi Maruyama and Kuniyoshi L. Sakai

Contents:

Supplementary Methods 2

Supplementary References 7

Supplementary Methods

Here, we provided a full description of the experimental conditions and the procedures used to acquire the data presented.

Participants

We analyzed the functional connectivity in 21 patients reported previously (Kinno et al. 2014); they were native Japanese speakers newly diagnosed as having a left frontal glioma (Table 2). The patients preoperatively performed the picture-sentence matching task, and underwent fMRI scans at the University of Tokyo, Komaba. They then underwent surgery at the Department of Neurosurgery, Tokyo Women's Medical University. All 21 patients met the following inclusion criteria: (i) right-handedness, (ii) no deficits in verbal/written communication or other cognitive abilities reported by the patients or physicians, (iii) no history of neurological or psychiatric disorders other than glioma and seizures, (iv) freedom from seizures with or without antiepileptic drugs, (v) no medical problems related to MRI acquisition, and (vi) completion of at least three fMRI runs without significant head movement. The laterality quotient of handedness was determined by the Edinburgh handedness inventory (Oldfield 1971). The antiepileptic drugs used were carbamazepine (400 mg/day), gabapentin (600 mg/day), phenobarbital (90-120 mg/day), phenytoin (200-300 mg/day), valproate acid (800-1200 mg/day), and zonisamide (200 mg/day).

The categorization criterion of each patient group was whether or not the glioma of a patient overlapped, at least partially on a voxel-by-voxel basis, with the regions identified by fMRI in our previous study (Kinno et al. 2008): the left LPMC shown in Figure 3 of that paper, and the left F3op/F3t shown in Figure 4 of that paper. The patients were divided into three groups based on the individual tumor locations in the normalized brain: the LPMC, F3, and Other groups; the number of patients was seven for each group without any selection. We also recruited 7 normal, age-matched, and right-handed participants [Normal group; age 25-43 years, 31 ± 5.9 (mean ± standard deviation)], thereby matching the sensitivity of the signal-to-noise ratio for MR signals in all tested groups. Written informed consent was obtained from each participant after the nature and possible consequences of the studies were explained. Approval for the experiments was obtained from the institutional review board of the University of Tokyo, Komaba, as well as that of the Tokyo Women's Medical University.

Lesion analyses

Each glioma was first identified on the normalized T1-weighted structural image, and the glioma boundary was semi-automatically determined using the 3D Fill tool in the MRIcroN software package (http://www.mccauslandcenter.sc.edu/mricro/mricron/), which generated a contiguous cluster of voxels defined by the intensity of the glioma itself. The boundary of each lesion, including brain edemas and abnormalities of perfusion, was confirmed with T2-weighted MR images taken at the Department of

Neurosurgery, Tokyo Women's Medical University. The absence of any skip lesions distant from a tumor was confirmed with ^{11}C-methionine, [^{18}F] fluorodeoxyglucose, and ^{11}C-choline positron emission tomography data (resolution = 4.8 × 4.8 × 4.25 mm^3) taken at the Chubu Medical Center for Prolonged Traumatic Brain Dysfunction (Minokamo City, Gifu, Japan). Lesion overlap maps, as well as each patient's activated regions, which were transformed back to the individual brains, were shown in our previous study (Kinno et al. 2014).

Stimuli

Each visual stimulus consisted of a picture with head symbols (circle, square, or triangle) at the top, and of an always grammatical sentence at the bottom (Kinno et al. 2014). For each stimulus, we chose two different head symbols. The sentences describing actions were written using a combination of the hiragana and kanji writing systems. In Japanese syntax, the grammatical relations ("subject, direct object, or indirect object" in linguistic terms) are first marked by grammatical particles (nominative, dative, or accusative), which in turn allow the assignment of semantic roles ("agent, experiencer, or patient" in linguistic terms, i.e., an agent who initiates the action, and an experiencer/patient who is affected by it), whereas passiveness is also marked in the verb morphology (*-areru*). We used four kinds of grammatical particles, which represent the syntactic information in Japanese: *-ga*, a nominative case marker; *-ni*, a dative case marker; *-o*, an accusative case marker; and *-to*, a coordinator (*and*). Two sets of Japanese verbs (six transitive verbs: *pull*, *push*, *scold*, *kick*, *hit*, and *call*; and six intransitive verbs: *lie*, *stand*, *walk*, *run*, *tumble*, and *cry*) were used, each of which, including the passive forms, had either four or five syllables. Note that the verb "*call*" is used only as a transitive verb in Japanese. There was no significant difference in frequency between the two sets of verbs ($t(10) = 0.7$, $P = 0.5$), according to the Japanese lexical database ("Nihongo-no Goitokusei" (Lexical Properties of Japanese), Nippon Telegraph and Telephone Corporation Communication Science Laboratories, Tokyo, Japan, 2003). The numbers of syllables and letters were strictly controlled among all conditions.

Using the same task, we tested two types of conditions with different sets of stimuli: Two-argument and One-argument conditions. Under the Two-argument conditions with an identical picture set, we tested three different sentence types: active, passive, and scrambled sentences. Scrambled sentences are perfectly normal, not only in Japanese but in German, Finnish, and other languages. Under the Two-argument conditions, each sentence ended with a transitive verb, and had two arguments (phrases associated with the predicate) with different grammatical relations and semantic roles. More specifically, the active, passive, and scrambled sentences corresponded to "subject and direct object" (agent and patient), "subject and indirect object" (experiencer and agent), and "direct object and subject" (patient and agent) types, respectively.

Under the One-argument condition, each sentence ended with an intransitive verb, and corresponded to a "double subjects" (double agents) type, which did not involve two-argument

relationships. Under the Two-argument conditions, the number of lines used in each picture except the head symbols was 14 ± 2.4, whereas under the One-argument condition, equally complex pictures (number of lines, 14 ± 2.5) were used. Under both conditions, half of the pictures depicted actions occurring from left to right, and the other half depicted actions occurring from right to left; head symbols were also counterbalanced for both sides. These pictures further excluded the involvement of pragmatic information about word use (e.g., "*An officer chases a thief*" is more acceptable than "*A thief chases an officer*"). There were 48 different stimuli (i.e., different combinations between pictures and sentences) for each of the Active, Passive, and Scrambled sentence conditions, as well as for the One-argument condition.

All stimuli were presented visually in yellow against a dark background. Each stimulus was presented for 5800 ms (intratrial interval) followed by a 200 ms blank interval. To minimize the effect of general memory demands, a whole sentence of a minimal length (i.e., two noun phrases and a verb) was visually presented for an ample time for the patients to respond (Kinno et al. 2014). The stimuli are thus more advantageous than sequentially presented stimuli that involve memorization. For fixation, a red cross was also shown at the center of the screen to initiate eye movements from the same fixed position, and the participants were instructed to return their eyes to this position after the response. The stimulus presentation and collection of behavioral data (error rates and reaction times) were controlled using the LabVIEW software and interface (National Instruments, Austin, TX). The participants wore earplugs and an eyeglass-like MRI-compatible display (resolution, 800 × 600; VisuaStim XGA, Resonance Technology Inc., Northridge, CA).

Task

In the picture-sentence matching task, the participants read a sentence silently and judged whether or not the action depicted in a picture matched the meaning of the sentence. They responded by pressing one of two buttons in a row (right for a matched pair, and left for a mismatched pair). Under the Two-argument conditions, all mismatched sentences were made by exchanging two symbols in the original sentences, e.g., "○ *pulls* Δ" instead of "Δ *pulls* ○". Under the One-argument condition, symbol-mismatched and action-mismatched sentences were presented equally often, requiring the sentences to be read completely. The participants underwent short practice sessions before task sessions to become fully familiarized with this task.

Using the same stimulus sets of pictures and letters presented under both Two-argument and One-argument conditions, participants also performed a nonlinguistic Control task, in which the participants judged whether or not two head symbols in the picture matched those among a letter string shown at the bottom, irrespective of their order. The letters in hiragana were jumbled without changing the head symbols and kanji, so that the letter string prevented even basic word recognition. General cognitive factors such as visual perception of the stimuli, matching, response selection, and motor responses were controlled by the Control task, and obviously by the One-argument condition as well.

A single run of the task sessions (306 s) contained 24 “test events” of the picture-sentence matching task (six times each for the Active, Passive, and Scrambled sentences, as well as for One-argument), with variable inter-trial intervals of one (6 s) or two (12 s) Control tasks. The order of the test events was pseudorandomized without repetition of the same condition to prevent any condition-specific strategy. A single run contained 27 trials of the Control task.

Considering the medical conditions of patients, we limited the MR scanning time to one hour with inter-task intervals of 5 min, restricting the number of in-scanner runs. All patients underwent three or four in-scanner runs. Normal participants were also tested under the same conditions, i.e., four in-scanner runs.

MRI data acquisition

The fMRI scans were conducted on a 1.5 T scanner (Stratis II, Premium; Hitachi Medical Corporation, Tokyo, Japan). We scanned 26 axial slices of 3-mm thickness with a 1-mm gap, covering the volume range of –40 to 63 mm from the anterior to posterior commissure line in the vertical direction, using an echo-planar imaging sequence (repetition time = 3 s, echo time = 50.5 ms, flip angle = 90°, field of view = 192×192 mm^2, resolution = 3×3 mm^2). In a single scan, we obtained 102 volumes following three dummy images, which allowed for the rise of the MR signals. After completion of the fMRI sessions, high-resolution T1-weighted images of the whole brain (192 axial slices, $0.75 \times 0.75 \times 1$ mm^3) were acquired from all participants with a radio frequency spoiled steady-state acquisition with a rewound gradient echo sequence (repetition time = 30 ms, echo time = 8 ms, flip angle = 60°, field of view = 192×192 mm^2).

fMRI data analyses

The fMRI data were analyzed in a standard manner using statistical parametric mapping (SPM8; Wellcome Trust Centre for Neuroimaging, http://www.fil.ion.ucl.ac.uk/spm/) software (Friston et al. 1995), implemented on MATLAB software (MathWorks, Natick, MA). The acquisition timing of each slice was corrected using the middle slice (the thirteenth slice chronologically) as a reference for the echo-planar imaging data. We realigned the time-series data to the first volume in each run, and removed runs that included data with a translation of > 2 mm in any of the three directions and with a rotation of > 1.4° around any of the three axes; these thresholds of head movement were empirically determined from our previous studies (Hashimoto and Sakai 2002; Suzuki and Sakai 2003; Kinno et al. 2008). For this reason, a single run was removed from three normal participants, two patients in the LPMC group, three patients in the F3 group, and two patients in the Other group, which was about 10% of all time points.

Each participant’s T1-weighted structural image was coregistered to the mean functional image generated during realignment. The coregistered structural image was spatially normalized to the standard brain space as defined by the Montreal Neurological Institute using the “unified segmentation" algorithm with medium regularization, which is a generative model that combines tissue segmentation (excluding

“other” tissues like a lesion, etc.), bias correction, and spatial normalization (Ashburner and Friston 2005). All of the normalized structural images were visually inspected and compared with the standard brain for the absence of any further deformation. A previous study has suggested that the unified models provide better and more reliable matching for brain images with focal lesions (Crinion et al. 2007). After spatial normalization, the resultant deformation field was applied to the realigned echo-planar imaging data in each run, which was resampled every 3 mm using seventh-degree B-spline interpolation. All normalized functional images were then smoothed by using an isotropic Gaussian kernel of 9 mm full-width at half maximum. Low-frequency noise was removed by high-pass filtering at 1/128 Hz. For creating an SPM design of each participant, hemodynamic responses induced by task trials were modeled with a boxcar function for 6 s from the onset of each stimulus, and the boxcar function was convolved with a hemodynamic response function. To minimize the effect of head movement, the six realignment parameters obtained from preprocessing were included as nuisance factors in a general linear model.

Functional connectivity analyses

The functional connectivity among multiple regions was assessed by a partial correlation method for the time-series fMRI data (Smith 2012). Using the MarsBaR-toolbox (http://marsbar.sourceforge.net/) for the SPM design, the time-series data were first averaged within a sphere of 6-mm radius centered at the local maximum of each region (Table 1). To discount the global differences of signal changes among the runs, the averaged time-series from each of all tested runs were normalized to those of the first run (an in-house MATLAB program). Using the concatenated runs of each participant, we calculated partial correlation coefficients for each of the time-series of two regions in question; we regressed out all the other nodes, before estimating the correlation between the two. The partial correlation coefficients were then averaged among the participants in each group to generate a partial correlation matrix.

For statistical evaluation, non-diagonal partial correlation coefficients (r-values) were first converted to Z-values by using the Fisher r-to-Z transformation. By using t-tests, we examined whether the mean Z-values *within* individual networks (e.g., a correlation between two regions in Network I) were significantly greater than those *between* any two of the networks (e.g., a correlation between a region in Network I and a region in Network II). To test any differences in mean Z-values between each patient group and Normal group, we used Dunnett tests. To examine any differences in mean Z-values among the pairs of two networks [e.g., (Networks III and I) vs. (Networks I and II)], we used Tukey-Kramer tests.

Supplementary References

Ashburner J, Friston KJ (2005) Unified segmentation. Neuroimage 26(3):839-851

Crinion J, Ashburner J, Leff A, Brett M, Price C, Friston K (2007) Spatial normalization of lesioned brains: Performance evaluation and impact on fMRI analyses. Neuroimage 37(3):866-875

Friston KJ, Holmes AP, Worsley KJ, Poline J-P, Frith CD, Frackowiak RSJ (1995) Statistical parametric maps in functional imaging: A general linear approach. Hum Brain Mapp 2:189-210

Hashimoto R, Sakai KL (2002) Specialization in the left prefrontal cortex for sentence comprehension. Neuron 35:589-597

Kinno R, Kawamura M, Shioda S, Sakai KL (2008) Neural correlates of noncanonical syntactic processing revealed by a picture-sentence matching task. Hum Brain Mapp 29(9):1015-1027

Kinno R, Ohta S, Muragaki Y, Maruyama T, Sakai KL (2014) Differential reorganization of three syntax-related networks induced by a left frontal glioma. Brain 137(4):1193-1212

Oldfield RC (1971) The assessment and analysis of handedness: The Edinburgh inventory. Neuropsychologia 9(1):97-113

Smith SM (2012) The future of FMRI connectivity. Neuroimage 62(2):1257-1266

Suzuki K, Sakai KL (2003) An event-related fMRI study of explicit syntactic processing of normal/anomalous sentences in contrast to implicit syntactic processing. Cereb Cortex 13(5):517-526
